# Skipping Breakfast Is Associated with Hypertension in Adults: A Meta-Analysis

**DOI:** 10.1155/2022/7245223

**Published:** 2022-03-03

**Authors:** Zishuo Li, Hongli Li, Qin Xu, Yanli Long

**Affiliations:** ^1^Administrative Office, Wuhan Asia Heart Hospital, Wuhan 430022, China; ^2^Coronary Care Unit, Wuhan Asia Heart Hospital, Wuhan 430022, China; ^3^Department of Cardiac Surgery, Wuhan Asia Heart Hospital, Wuhan 430022, China

## Abstract

Previous studies evaluating the association between skipping breakfast and hypertension in adult population showed inconsistent results. We performed a meta-analysis to systematically evaluate the association. Observational studies which evaluated the relationship between skipping breakfast and hypertension in adult population with multivariate analyses were identified by systematic search of PubMed, Embase, and Web of Science databases. A random-effect model which incorporated the potential intrastudy heterogeneity was used for the meta-analysis. A total of six observational studies with 14189 adults were included, and 3577 of them were breakfast skippers. Pooled results showed that skipping breakfast was independently associated with hypertension in these populations (adjusted odds ratio (OR): 1.20, 95% confidence interval: 1.08 to 1.33, *P* < 0.001) with no significant heterogeneity (*I*^2^ = 0%). Sensitivity by excluding one study at a time showed consistent results (OR: 1.18 to 1.22, all *P* <0.01). Subgroup analyses showed that the association between skipping breakfast and hypertension in adults was consistent in the general population and in patients with type 2 diabetes, in studies from different countries, in cohort and cross-sectional studies, in breakfast skippers defined as taking breakfast ≤3 days/week and as self-reported habitual breakfast skipping, and in studies with and without adjustment of body mass index (*P*for subgroup difference, all *P*>0.10). In conclusion, skipping breakfast is associated with hypertension in the adult population.

## 1. Background

Hypertension is a prevalent risk factor of cardiovascular diseases, particularly in elderly population and in the developing countries [1–3]. Reducing the incidence of hypertension in global population is important for public health, which highlights the significance of identifying possible modifiable risk factors for hypertension [4, 5]. Poor lifestyles have been associated with the pathogenesis of various cardiometabolic disorders, including hypertension [6, 7]. Accumulating evidence from epidemiological studies showed that skipping breakfast is related to increased risks of obesity [8–10], type 2 diabetes mellitus (T2DM) [11, 12], cardiovascular diseases [13–15], and all-cause mortality [14, 15]. However, studies evaluating the association between skipping breakfast and hypertension in the adult population showed inconsistent results [16–21]. Some studies suggested that skipping breakfast may be an independent risk factor for hypertension in adults [18–20], while others did not [16, 17, 21]. Moreover, a recent meta-analysis of randomized controlled trials (RCTs) including 425 participants showed that skipping breakfast with an average duration of 8.6 weeks did not significantly change the blood pressure (BP) [22], which further suggested the uncertainty regarding the relationship between skipping breakfast and hypertension. Accordingly, in this study, we performed a meta-analysis to systematically evaluate the association between skipping breakfast and hypertension in adult participants.

## 2. Methods

The meta-analysis was performed in accordance with the MOOSE (Meta-Analysis of Observational Studies in Epidemiology) [23] and Cochrane's Handbook [24] guidelines.

### 2.1. Literature Search

Studies were identified via systematic search of electronic databases of PubMed and Embase via the following terms: (1) “breakfast” OR “morning meal” OR “eating patterns” OR “meal frequency” OR “skipping meals” and (2) “hypertension” OR “blood pressure” OR “hypertensive.” The search strategies were developed based on previous meta-analyses of “skipping breakfast” [8] and “hypertension” [25], respectively, which have been validated in these previous meta-analyses. The search was limited to human studies published in English. The reference lists of related original and review articles were also analyzed using a manual approach. The final literature search was performed on September 10, 2021.

### 2.2. Study Selection

The inclusion criteria for the studies were as follows: (1) observational studies published as full-length articles; (2) included adult population; (3) evaluated the association between skipping breakfast and hypertension; and (4) reported the relative risk for this association after adjustment of potential confounding factors. The definition of skipping breakfast was consistent with the criteria of the included studies. Diagnosis of hypertension was in accordance with the criteria applied in the included studies, which was generally defined as systolic BP ≥ 140 mmHg and diastolic BP ≥ 90 mmHg or on treatment of antihypertensive medications. Reviews, editorials, studies in children and adolescents, studies without information about breakfast skipping, studies irrelevant to the aim of the current meta-analysis, and studies with univariate analysis were excluded.

### 2.3. Data Extraction and Quality Evaluation

Literature search, data extraction, and quality assessment of the included studies were performed according to the predefined inclusion criteria. Discrepancies were resolved by consensus. The extracted data included the following: (1) name of first author, publication year, and country where the study was performed; (2) study design characteristics; (3) participant characteristics, including health status, sample size, age, sex, and body mass index (BMI); (4) exposure characteristics, including definition of breakfast skipping and number of breakfast skippers included; (5) follow-up durations for cohort studies; (6) methods for hypertension evaluation and the number of patients with hypertension; and (7) confounding factors adjusted in the multivariate analyses. The quality of each study was evaluated using the Newcastle-Ottawa Scale [26] which ranges from 1 to 9 stars and judges each study regarding three aspects: the selection of the study groups; the comparability of the groups; and the ascertainment of the outcome of interest.

### 2.4. Statistical Analyses

We used odds ratios (ORs) and their corresponding 95% confidence intervals (CIs) as the general measure for the association between breakfast skipping and hypertension in the adult population. Data of ORs and their corresponding stand errors (SEs) were calculated from 95% CIs or *P* values and were logarithmically transformed to stabilize variance and normalize the distribution [24]. Cochrane's *Q* test and estimation of *I*^2^ statistic were used to evaluate the heterogeneity among the included observational studies [27]. Significant heterogeneity was considered if *I*^2^ > 50%. We used a random-effect model to synthesize the OR data because this model is considered as a more generalized method which incorporates the potential heterogeneity among the included studies [24]. Sensitivity analyses, by omitting one individual study at a time, were performed to test the robustness of the results [28]. Predefined subgroup analyses were performed to evaluate the influences of study characteristics on the outcome, such as population characteristics, study design, country of the study, definition of breakfast skipper, adjustment of BMI, and quality scores. The potential publication bias was assessed by visual inspection of the symmetry of the funnel plots as well as Egger's regression asymmetry test [29]. We used RevMan (Version 5.1; Cochrane Collaboration, Oxford, UK) and STATA software for the meta-analysis and statistics.

## 3. Results

### 3.1. Literature Search

**Figure 1:**
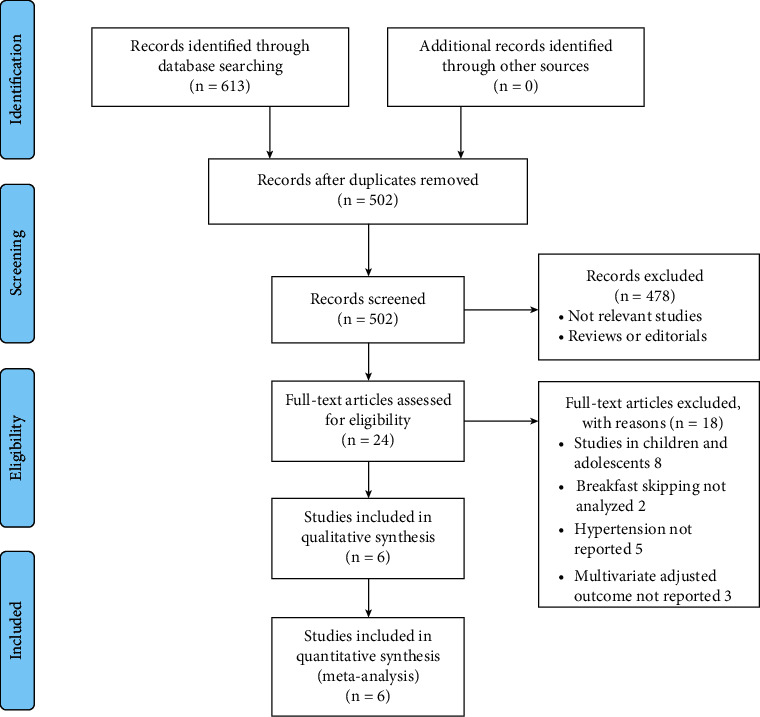
Flowchart of database search and study identification.

The process of database search is summarized in Figure 1. In brief, 502 articles were found via an initial literature search of the PubMed, Embase, and Web of Science databases after excluding the duplications. Among them, 478 were excluded through screening of the titles and abstracts mainly because they were not relevant to the purpose of the meta-analysis. Subsequently, 24 potential relevant records underwent full-text review. Of these, 18 were further excluded for the reasons listed in Figure 1. Finally, six observational studies were included for the meta-analysis [16–21].

### 3.2. Study Characteristics and Quality Evaluation

The characteristics of the included studies are summarized in Table 1. Overall, one prospective cohort study [18] and five cross-sectional studies [16, 17, 19–21] with 14189 adults were included. The studies were published between 2011 and 2021 and performed in Korea [16, 19], Ghana [20], and the United States [17, 18, 21], respectively. Community adult population was included in three studies [17–19], apparently healthy employees were included in two studies [16, 21], and the other one included patients with T2DM [20]. Evaluation of skipping breakfast was based on questionnaires in all of the included studies, and breakfast skippers were defined as participants who took breakfast ≤3 days/week in three studies [16, 18, 20] and as self-reported habitual breakfast skippers for the other three studies [17, 19, 21]. A total of 3577 participants were breakfast skippers at baseline. Validation of the hypertension outcome was performed by clinical examination by trained research members. Age, sex, BMI, smoking status, and other potential confounding factors were generally adjusted to varying degrees when the association between skipping breakfast and hypertension was reported. The NOS scores of the included studies ranged from eight to nine, indicating generally good study quality.

### 3.3. Association between Skipping Breakfast and Hypertension

**Figure 2:**
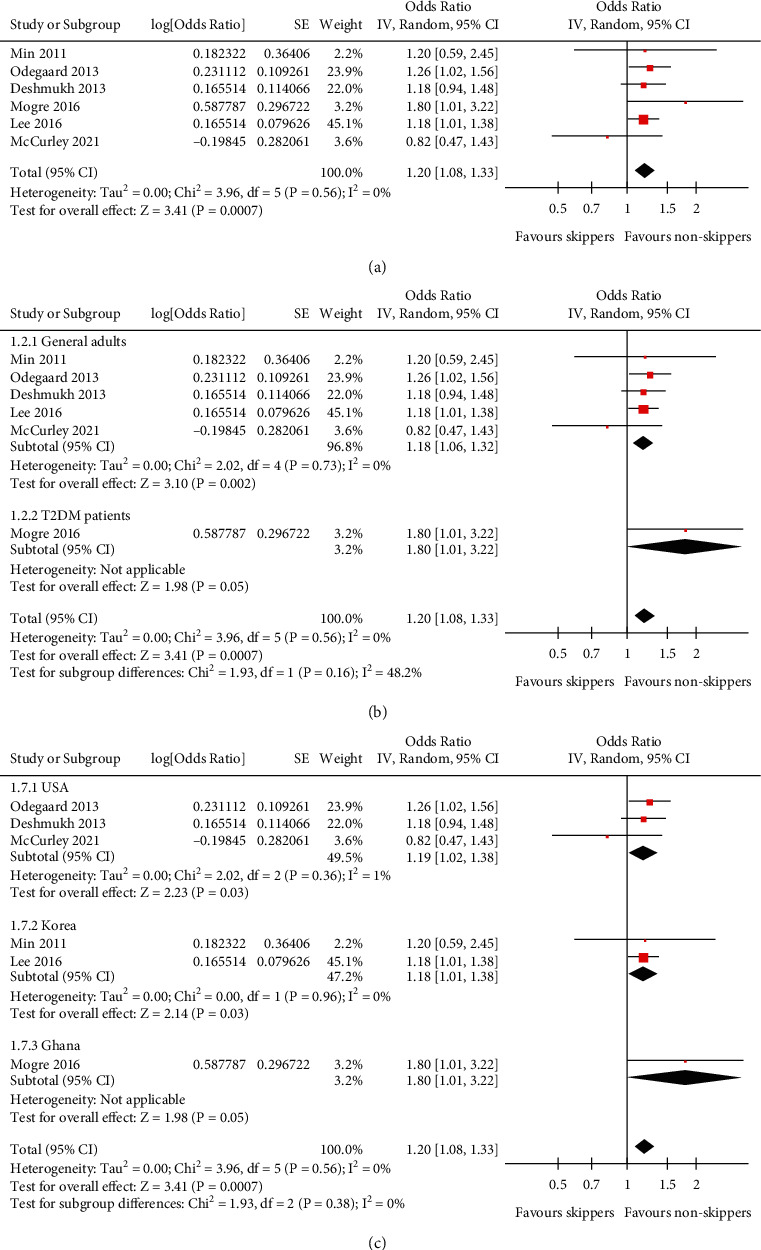
Forest plots for the meta-analysis of the association between skipping breakfast and hypertension in the adult population. (a) Results of overall meta-analysis. (b) Subgroup analysis according to the characteristics of the included participants. (c) Subgroup analysis according to the country of the study.

**Figure 3:**
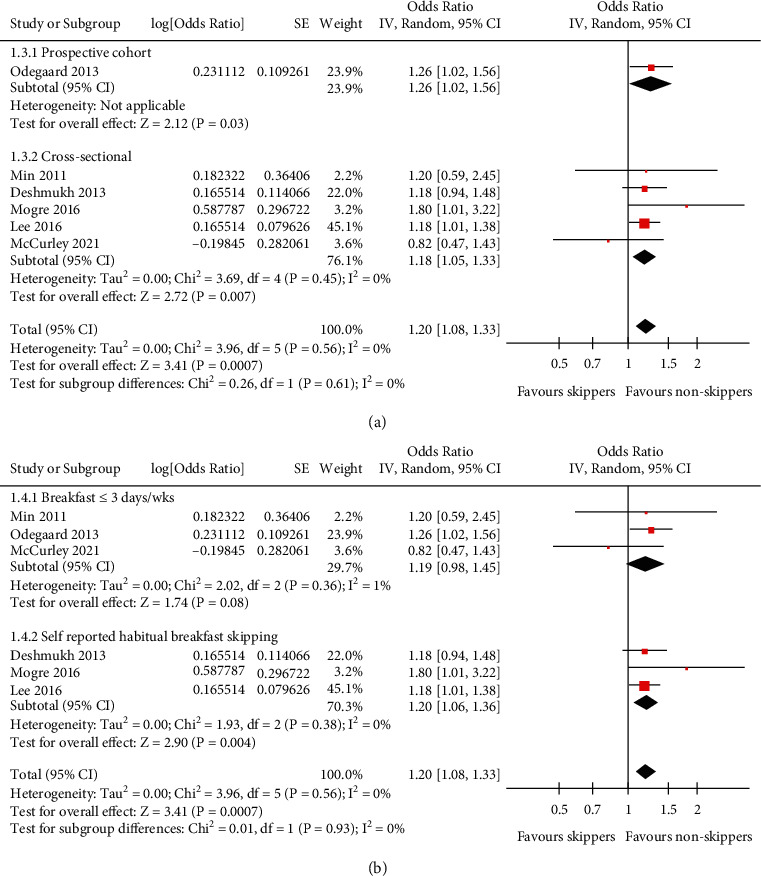
Forest plots for the subgroup analyses of the association between skipping breakfast and hypertension in the adult population. (a) Subgroup analysis according to study design. (b) Subgroup analysis according to the definition of skipping breakfast.

**Figure 4:**
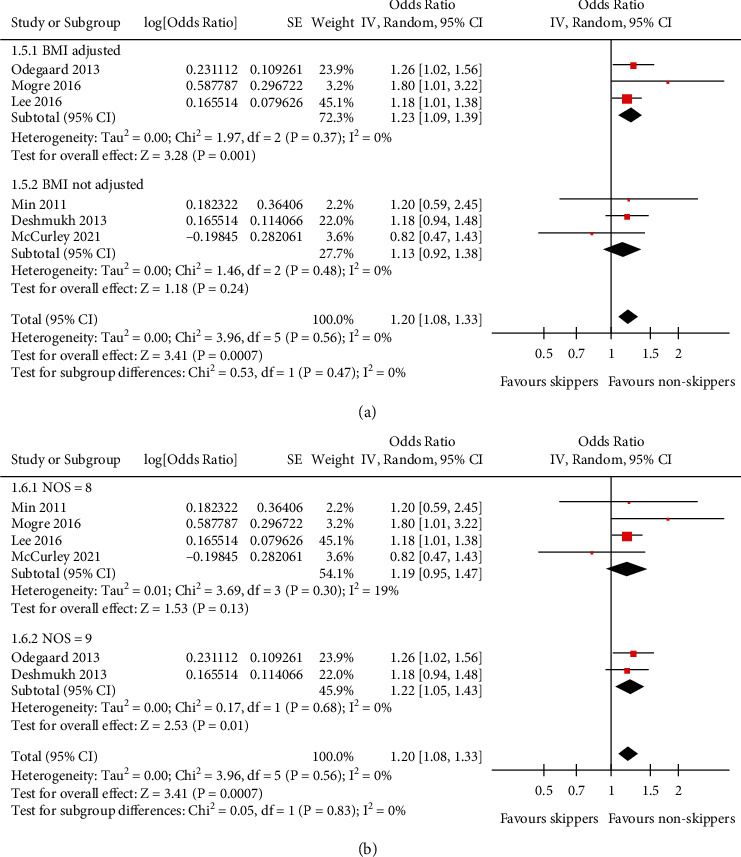
Forest plots for the subgroup analyses of the association between skipping breakfast and hypertension in the adult population. (a) Subgroup analysis according to the adjustment of BMI. (b) Subgroup analysis according to the quality scores.

Pooled results with a random-effect model showed that compared to people who ate breakfast regularly, people skipping breakfast were independently associated with higher odds of hypertension (adjusted OR: 1.20, 95% CI: 1.08 to 1.33, *P* < 0.001; Figure 2(a)) with no statistically significant heterogeneity (*P* for Cochrane's *Q* test = 0.56, *I*^2^ = 0%). Sensitivity by excluding one study at a time showed consistent results (OR: 1.18 to 1.22, all *P* <0.01; Table 2). Subgroup analyses showed that the association between skipping breakfast and hypertension in adults was consistent in general population and in patients with T2DM (*P* for subgroup difference = 0.16; Figure 2(b)), in studies performed in the United States, Korea, and Ghana (*P* for subgroup difference = 0.38; Figure 2(c)), in prospective cohort and cross-sectional studies (*P* for subgroup difference = 0.61; Figure 3(a)), in breakfast skippers defined as taking breakfast ≤3 days/week and as self-reported habitual breakfast skipping (*P* for subgroup difference = 0.93; Figure 3(b)), in studies with and without the adjustment of BMI *P* for subgroup difference = 0.47; Figure 4(a)), and in studies with quality scores of eight and nine (*P* for subgroup difference = 0.83, Figure 4(b)).

### 3.4. Publication Bias

**Figure 5:**
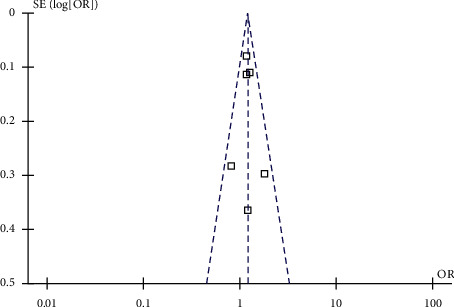
Funnel plots for the publication bias underlying meta-analysis of the association between skipping breakfast and hypertension in the adult population.

The funnel plots regarding the association between skipping breakfast and hypertension in the adult population are shown in Figure 5. The funnel plots were symmetric on visual inspection, suggesting a low risk of publication bias. Egger's regression tests showed consistent results (*P*=0.852).

## 4. Discussion

In this meta-analysis, we pooled the results of six available observational studies, and the results showed that skipping breakfast is associated with a moderately increased risk of hypertension in the adult population. Further results of sensitivity analyses showed the robustness of the findings which were not primarily driven by either of the included studies. Moreover, results of subgroup analyses showed that the findings of the meta-analyses were not significantly affected by study characteristics, such as the source of the participants, country of the study, study design, definition of breakfast skippers, adjustment of BMI, and quality scores of the included studies. Taken together, these results indicated that skipping breakfast was associated with hypertension in the adult population, and the association seemed to be independent of previously proposed confounding factors such as obesity.

To the best of our knowledge, this study may be the first meta-analysis summarizing the current evidence for the association between breakfast skipping and hypertension in the adult population. Our meta-analysis has several methodological strengths which should be noted before the interpretation of the results. Only studies with multivariate analysis were included in this meta-analysis, which therefore could provide an independent association between skipping breakfast and hypertension. Moreover, comprehensive sensitivity and subgroup analyses were performed, and the consistent results suggested the stability and robustness of the findings of the meta-analysis. Results of a meta-analysis showed that skipping breakfast was associated with hypertension, which may suggest that skipping breakfast is a modifiable risk factor for hypertension in the adult population. Several mechanisms underlying the association between skipping breakfast and hypertension have been proposed. Firstly, skipping breakfast may cause hunger sensations and overeating later in the day, leading to insulin resistance, overweight, and obesity [30]. Indeed, previous studies have consistently shown that skipping breakfast is associated with being overweight and obese [8–10], which may be a key mechanism underlying the association between skipping breakfast and increased cardiometabolic risks [31]. Since overweight and obesity have been well acknowledged as major risk factors for hypertension [32], it should be clarified if the association between skipping breakfast and hypertension exists. However, results of subgroup analysis showed that the association between skipping breakfast and hypertension was consistent in studies with and without adjustment of BMI, suggesting that the association could not be fully explained by the factor of overweight/obesity. In addition, skipping breakfast may be a behavioral marker of a cluster of unhealthy lifestyles, such as poor dietary habit, low physical activity, and irregular sleeping [33, 34], all of which may expose the participants to a higher risk of hypertension. Besides, habitual breakfast skipping is associated with higher levels of systematic inflammatory markers such as C-reactive protein [35] and glycoprotein acetyl [36], which may suggest chronic inflammation as a possible molecular basis for the association between skipping breakfast and hypertension. Studies are warranted in the future to clarify the mechanisms underlying the association between breakfast skipping and hypertension. Nevertheless, studies are also needed to determine whether long-term regular breakfast eating in former breakfast skippers is associated with improved cardiometabolic risk profiles, including hypertension.

Our study also has limitations. Firstly, the number of available studies for the meta-analysis is limited. We were unable to determine a dose-response association between skipping breakfast and hypertension according to the frequency and duration of the breakfast skipping habits. Besides, the possible influences of participant characteristics on the association of interest could not be determined based on this meta-analysis, such as age and sex. Moreover, we were unable to determine the ethnic differences in breakfasts on the results of the meta-analyses because none of the included studies reported the outcome according to the ethnicity of the included population. However, a subgroup analysis based on country of the study showed a consistent association between skipping breakfast and hypertension in studies from the USA, Korea, and Ghana. In addition, although the results of the meta-analysis were based on multivariate analyses, we could not exclude the possible influence of residual factors which may confound the association, such as dietary components. Finally, a causative relationship between skipping breakfast and hypertension could not be derived from this meta-analysis because it is based on observational studies.

## 5. Conclusions

In conclusion, the results of this meta-analysis suggested that skipping breakfast is associated with a moderately increased risk of hypertension in the adult population. Future studies are needed to determine whether long-term regular breakfast eating in former breakfast skippers is associated with improved cardiometabolic risk profiles, including hypertension.

## Figures and Tables

**Table 1:** Characteristics of the included observational studies.

Study	Country	Design	Participant characteristics	Sample size	Age	Male	Mean BMI	Definition of breakfast skippers	Number of breakfast skippers	Follow-up duration	Number of hypertensive patients	Variables adjusted	Quality score
					years	%	kg/m^2^			years			
Min 2011	Korea	CS	Apparently healthy employees	415	30–50	28.4	23.2	Taking breakfast ≤3 days/week	44	NA	NR	Age and sex	8
Odegaard 2013	USA	PC	Community young adults	3598	25∼37	45.5	26.7	Taking breakfast ≤3 days/week	1556	18	1003	Age, study center, race, sex, education, cigarette smoking, physical activity, alcohol consumption, fast food restaurant use, dietary quality score, and BMI	9
Deshmukh 2013	USA	CS	Community young adults	5316	20∼39	57.7	27.6	Self-reported habitual breakfast skipping	1277	NA	1313	Age, gender, ethnicity, poverty income ratio, smoking status, alcohol consumption, physical activity, energy intake, and marital status	9
Mogre 2016	Ghana	CS	T2DM patients	378	20∼70	34.9	26.8	Self-reported habitual breakfast skipping	88	NA	256	Age, sex, duration of DM, marriage, education, BMI, and family history of DM	8
Lee 2016	Korea	CS	Community adults	3880	>20	40.5	23.7	Self-reported habitual breakfast skipping	510	NA	1004	Age, sex, DM, regular exercise, current smoking, BMI, WC, and RBC count	8
McCurley 2021	USA	CS	Hospital employees	602	20∼75	21	NR	Taking breakfast ≤3 days/week	102	NA	125	Age, sex, race, ethnicity, education, shift work status, marital status, household size, job type, 1-year weight goal, and number of items purchased	8

BMI, body mass index; NR, not reported; NA, not applicable; CS, cross-sectional; PC, prospective cohort; DM, diabetes mellitus; T2DM, type 2 DM; RBC, red blood cells; WC, waist circumference.

**Table 2:** Sensitivity analyses.

Study excluded	OR (95% CI)	*I* ^2^	*P* for Cochrane's *Q* test	*P* for overall effect
Min 2011	1.20 [1.08, 1.33]	0%	0.41	<0.001
Odegaard 2013	1.18 [1.05, 1.33]	0%	0.45	0.007
Deshmukh 2013	1.21 [1.07, 1.36]	0%	0.42	0.002
Mogre 2016	1.18 [1.06, 1.32]	0%	0.73	0.002
Lee 2016	1.22 [1.06, 1.40]	0%	0.42	0.007
McCurley 2021	1.22 [1.09, 1.35]	0%	0.72	<0.001

OR, odds ratio; CI, confidence interval.

## Data Availability

The data used to support the findings of this study are available from the corresponding author upon request.

## References

[1] Mills K. T., Stefanescu A., He J. (2020). The global epidemiology of hypertension. *Nature Reviews Nephrology*.

[2] Oliveros E., Patel H., Kyung S. (2020). Hypertension in older adults: assessment, management, and challenges. *Clinical Cardiology*.

[3] Virani S. S., Alonso A., Aparicio H. J. (2021). Heart disease and stroke statistics-2021 update: a report from the American heart association. *Circulation*.

[4] Campbell N. R. C., Niebylski M. L. (2014). Prevention and control of hypertension. *Current Opinion in Cardiology*.

[5] Ghaffari S., Roshanravan N. (2020). The role of nutraceuticals in prevention and treatment of hypertension: an updated review of the literature. *Food Research International*.

[6] Ozemek C., Tiwari S., Sabbahi A., Carbone S., Lavie C. J. (2020). Impact of therapeutic lifestyle changes in resistant hypertension. *Progress in Cardiovascular Diseases*.

[7] Valenzuela P. L., Carrera-Bastos P., Gálvez B. G. (2021). Lifestyle interventions for the prevention and treatment of hypertension. *Nature Reviews Cardiology*.

[8] Ardeshirlarijani E., Namazi N., Jabbari M. (2019). The link between breakfast skipping and overweigh/obesity in children and adolescents: a meta-analysis of observational studies. *Journal of Diabetes and Metabolic Disorders*.

[9] Horikawa C., Kodama S., Yachi Y. (2011). Skipping breakfast and prevalence of overweight and obesity in Asian and Pacific regions: a meta-analysis. *Preventive Medicine*.

[10] Ma X., Chen Q., Pu Y. (2020). Skipping breakfast is associated with overweight and obesity: a systematic review and meta-analysis. *Obesity Research & Clinical Practice*.

[11] Bi H., Gan Y., Yang C., Chen Y., Tong X., Lu Z. (2015). Breakfast skipping and the risk of type 2 diabetes: a meta-analysis of observational studies. *Public Health Nutrition*.

[12] Ballon A., Neuenschwander M., Schlesinger S. (2019). Breakfast skipping is associated with increased risk of type 2 diabetes among adults: a systematic review and meta-analysis of prospective cohort studies. *Journal of Nutrition*.

[13] Takagi H., Hari Y., Nakashima K., Kuno T., Ando T. (2019). Meta-analysis of relation of skipping breakfast with heart disease. *The American Journal of Cardiology*.

[14] Ofori-Asenso R., Owen A. J., Liew D. (2019). Skipping breakfast and the risk of cardiovascular disease and death: a systematic review of prospective cohort studies in primary prevention settings. *J Cardiovasc Dev Dis*.

[15] Chen H., Zhang B., Ge Y. (2020). Association between skipping breakfast and risk of cardiovascular disease and all cause mortality: a meta-analysis. *Clinical Nutrition*.

[16] Min C., Noh H., Kang Y.-S. (2011). Skipping breakfast is associated with diet quality and metabolic syndrome risk factors of adults. *Nutrition Research and Practice*.

[17] Deshmukh-Taskar P., Nicklas T. A., Radcliffe J. D., O’Neil C. E., Liu Y. (2013). The relationship of breakfast skipping and type of breakfast consumed with overweight/obesity, abdominal obesity, other cardiometabolic risk factors and the metabolic syndrome in young adults. The National Health and Nutrition Examination Survey (NHANES): 1999-2006. *Public Health Nutrition*.

[18] Odegaard A. O., Jacobs D. R., Steffen L. M., Van Horn L., Ludwig D. S., Pereira M. A. (2013). Breakfast frequency and development of metabolic risk. *Diabetes Care*.

[19] Lee T. S., Kim J. S., Hwang Y. J., Park Y. C. (2016). Habit of eating breakfast is associated with a lower risk of hypertension. *Journal of Lifestyle Medicine*.

[20] Mogre V., Apala P., Nsoh J. A., Wanaba P. (2016). Adiposity, hypertension and weight management behaviours in Ghanaian type 2 diabetes mellitus patients aged 20-70 years. *Diabetes & metabolic syndrome*.

[21] McCurley J. L., Levy D. E., Dashti H. S. (2021). Association of employees’ meal skipping patterns with workplace food purchases, dietary quality, and cardiometabolic risk: a secondary analysis from the ChooseWell 365 trial. *Journal of the Academy of Nutrition and Dietetics*.

[22] Bonnet J. P., Cardel M. I., Cellini J., Hu F. B., Guasch‐Ferré M. (2020). Breakfast skipping, body composition, and cardiometabolic risk: a systematic review and meta-analysis of randomized trials. *Obesity*.

[23] Stroup D. F., Berlin J. A., Morton S. C. (2000). Meta-analysis of observational studies in epidemiology: a proposal for reporting. Meta-analysis of Observational Studies in Epidemiology (MOOSE) group. *JAMA*.

[24] Higgins J., Green S. (2011). *Cochrane Handbook for Systematic Reviews of Interventions Version 5.1.0*.

[25] Wang Y., Yang W., Jiang X. (2021). Association between triglyceride-glucose index and hypertension: a meta-analysis. *Frontiers in Cardiovascular Medicine*.

[26] Wells G. A., Shea B., O’Connell D. (2010). The Newcastle-Ottawa Scale (NOS) for assessing the quality of nonrandomised studies in meta-analyses. http://www.ohri.ca/programs/clinical_epidemiology/oxford.asp.

[27] Higgins J. P. T., Thompson S. G. (2002). Quantifying heterogeneity in a meta-analysis. *Statistics in Medicine*.

[28] Patsopoulos N. A., Evangelou E., Ioannidis J. P. (2008). Sensitivity of between-study heterogeneity in meta-analysis: proposed metrics and empirical evaluation. *International Journal of Epidemiology*.

[29] Egger M., Smith G. D., Schneider M., Minder C. (1997). Bias in meta-analysis detected by a simple, graphical test. *BMJ*.

[30] Uzhova I., Mullally D., Peñalvo J. L., Gibney E. R. (2018). Regularity of breakfast consumption and diet: insights from national adult nutrition survey. *Nutrients*.

[31] Ibáñez B., Fernández-Alvira J. M. (2019). Breakfast is a marker for cardiovascular risk prediction. *Journal of the American College of Cardiology*.

[32] Dwivedi A. K., Dubey P., Cistola D. P., Reddy S. Y. (2020). Association between obesity and cardiovascular outcomes: updated evidence from meta-analysis studies. *Current Cardiology Reports*.

[33] Faris M. E., Vitiello M. V., Abdelrahim D. N. (2021). Eating habits are associated with subjective sleep quality outcomes among university students: findings of a cross-sectional study. *Sleep and Breathing*.

[34] Sila S., Ilić A., Mišigoj-Duraković M., Sorić M., Radman I., Šatalić Z. (2019). Obesity in adolescents who skip breakfast is not associated with physical activity. *Nutrients*.

[35] Zhu S., Cui L., Zhang X. (2021). Habitually skipping breakfast is associated with chronic inflammation: a cross-sectional study. *Public Health Nutrition*.

[36] Guinter M. A., Campbell P. T., Patel A. V., McCullough M. L. (2019). Irregularity in breakfast consumption and daily meal timing patterns in association with body weight status and inflammation. *British Journal of Nutrition*.

